# Interaction of Negative Bias Instability and Self-Heating Effect on Threshold Voltage and SRAM (Static Random-Access Memory) Stability of Nanosheet Field-Effect Transistors

**DOI:** 10.3390/mi15030420

**Published:** 2024-03-21

**Authors:** Xiaoming Li, Yali Shao, Yunqi Wang, Fang Liu, Fengyu Kuang, Yiqi Zhuang, Cong Li

**Affiliations:** 1 School of Microelectronics, Xidian University, Xi’an 710071, China; 2 Beijing Smartchip Microelectronics Technology Company Limited, Beijing 100089, China

**Keywords:** NSFET, negative bias instability (NBTI), self-heating effect (SHE), nanosheet width, reliability, technology computer-aided design (TCAD)

## Abstract

In this paper, we investigate the effects of negative bias instability (NBTI) and self-heating effect (SHE) on threshold voltage in NSFETs. To explore accurately the interaction between SHE and NBTI, we established an NBTI simulation framework based on trap microdynamics and considered the influence of the self-heating effect. The results show that NBTI weakens the SHE effect, while SHE exacerbates the NBTI effect. Since the width of the nanosheet in NSFET has a significant control effect on the electric field distribution, we also studied the effect of the width of the nanosheet on the NBTI and self-heating effect. The results show that increasing the width of the nanosheet will reduce the NBTI effect but will enhance the SHE effect. In addition, we extended our research to the SRAM cell circuit, and the results show that the NBTI effect will reduce the static noise margin (SNM) of the SRAM cell, and the NBTI effect affected by self-heating will make the SNM decrease more significantly. In addition, our research results also indicate that increasing the nanosheet width can help slow down the NBTI effect and the negative impact of NBTI on SRAM performance affected by the self-heating effect.

## 1. Introduction

With the development of Moore’s law, MOSFETs’ size reduction is becoming the most critical issue in the integrated circuit industry [1,2,3,4]. Short-channel effects (SCEs) and reliability are the main factors limiting size reduction [5,6]. To overcome SCEs, multi-gate devices have replaced traditional planar MOSFETs due to their better gate controllability, such as FinFETs used in the 22 nm CMOS technology node and beyond [7]. However, due to technical difficulties and the intensification of SCEs, nanosheet field-effect transistors (NSFETs) with their GAA structure, providing better gate control capabilities and more flexible current regulation capabilities due to the adjustable width, have replaced FinFETs as the ideal transistor for the 5 nm node and beyond [8,9]. 

Current research on NSFETs mainly focuses on improving electrical and radio frequency characteristics by optimizing the device structure [10,11]. Recently, researchers have begun to pay attention to the impact of reliability issues on NSFETs [12,13]. In particular, the NBTI effect is the most severe impact on MOSFET devices [14]. Until now, many papers have studied the NBTI effect of NSFETs [15,16,17].

An NSFET is a GAA structure with poor heat dissipation compared to bulk-FinFET devices with bulk silicon substrates [18]. Furthermore, to improve the mobility of P-type NSFETs, NSFET devices with advanced technology often use SiGe in the source and drain, but the thermal conductivity of SiGe is low, which aggravates the self-heating effect in NSFETs [19,20,21].

For P-type NSFETs, the intensification of the self-heating effect not only alters the device’s electrical characteristics but also significantly impacts the NBTI effect in PMOS devices [22,23]. Researchers have recently conducted relevant studies on the coupling effect of NBTI and SHE. Recently, researchers have studied the impact of NBTI and SHE in FinFETs [24], and some researchers have adopted a relatively simple BTI model to study the effects of NBTI and SHE on NW FETs. Although they did not start from a trap dynamics perspective, their study still provides valuable insights [25]. Although some people have studied how to decouple NBTI and HCI effects under the influence of self-heating effects, they have yet to explore the interrelationship between NBTI and self-heating effects in detail [26]. Considering the importance of NSFETs and the severe impact of the self-heating effect on NBTI in P-type NSFETs, it is necessary to conduct in-depth research on this issue.

In addition, in the NSFET structure, the electric field distribution is not uniform [27], which leads to the aggravation of the influence on the NBTI effect [16]. Considering these factors, based on previous studies, the relationship between the structural parameters of NSFETs and the NBTI effect also needs to be analyzed deeply. Furthermore, according to the International Technology Roadmap for Semiconductors (ITRS) report, there is a very strong demand for stable thermal/electrical reliability for circuit features such as SRAM and ring oscillators (ROs) [28]. Since SRAM usually comprises multiple CMOS transistors, SRAMs made from stacked NSFETs may have thermal issues [29].

In this paper, to study accurately the impact of SHE on the NBTI effect of NSFETs, we built a simulation framework for NBTI in TCAD based on trap microscopic dynamics while considering the influence of the self-heating effect. In addition, the width of the nanosheet is adjusted to improve the uniformity of the electric field distribution to mitigate the NBTI effect and its interaction with the self-heating effect. We also extend our study to SRAM cell circuits to analyze the impact of device reliability on the stability of SRAM circuits.

The paper is summarized as follows: Section 2 describes the device structure, the simulation setup, and the NBTI simulation framework. Section 3 discusses the interaction between NBTI and self-heating effects in NSFETs and their impact on NSFET-based SRAM circuits. Finally, in Section 4, conclusions and important points are presented.

## 2. Device Structure and Simulation Setup

### 2.1. Device Structure

**Figure 1:**
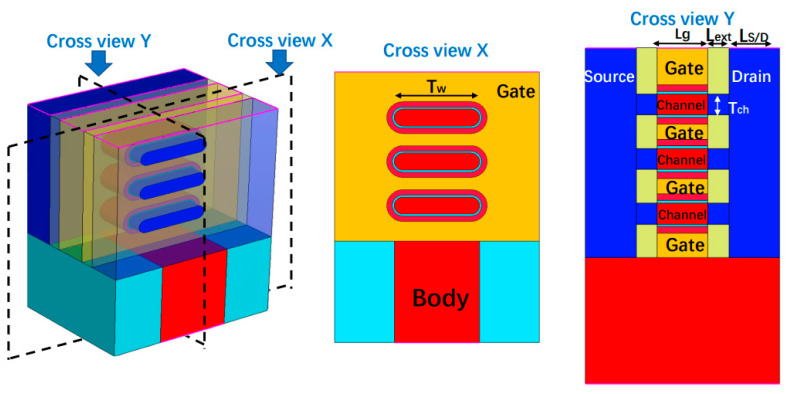
From left to right: the 3-D structure of the 5 nm node three-stack nanosheet FET, cross-view X, and cross-view Y with channel details.

Figure 1 shows the three-dimensional structure and cross-section of the NSFET device. The design of this NSFET device follows the 2015 International Technology Roadmap for Semiconductors (ITRS) [30]. The gate length (Lg) is 12 nm, the extension length (Lext) is 5 nm, the channel width (Tw) is 25 nm, and the entire channel thickness (Tch) is 5 nm, with an S/D length of 12 nm. Regarding the oxide layer, the SiO_2_ thickness (κ ≈ 3.9) is 0.6 nm, the HfO_2_ thickness (κ ≈ 22) is 1.69 nm, and the equivalent oxide layer thickness (EOT) is 0.9 nm. In the S/D region, the boron doping concentration is 1 × 10^20^, while in the channel region, the phosphorus doping concentration is 1 × 10^16^. All these structure parameters are specified in Table 1.

### 2.2. TCAD Simulation

**Figure 2:**
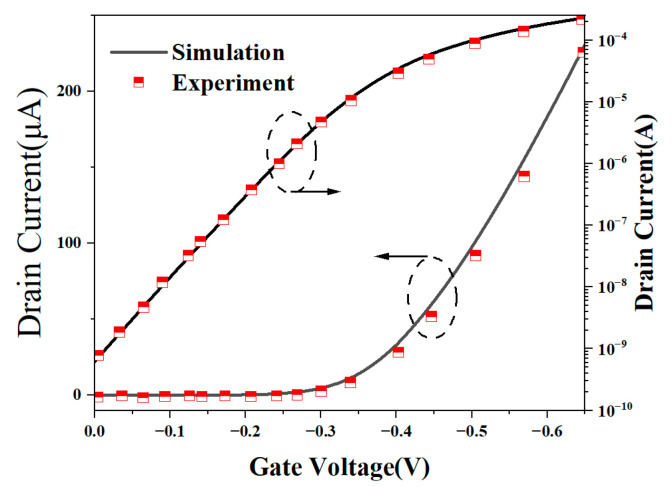
Calibrated Id–Vg curve of the nanosheet GAA transistor with data from [31].

We simulated the NSFET using the TCAD (Sentaurus TCAD 2021) [16]. We employed the Lombardi model to account for mobility degradation caused by impurity scattering and intercarrier scattering. Due to the influence of the thickness of the sheet, the thin-layer mobility model is used. Additionally, to consider the quantum effects related to carrier density and density gradient, we incorporated Fermi–Dirac statistics and quantum potentials. The band narrowing model and the Shockley–Read–Hall (SRH) composite model were also included. To simulate the tunneling process, we utilized the Hurkx BTBT model. Figure 2 shows the calibration results of the transfer characteristics of NSFETs with all-silicon channels, which are consistent with the experimental data. It should be noted that the experimental data are extracted from [31].

### 2.3. NBTI Simulation Framework

**Figure 3:**
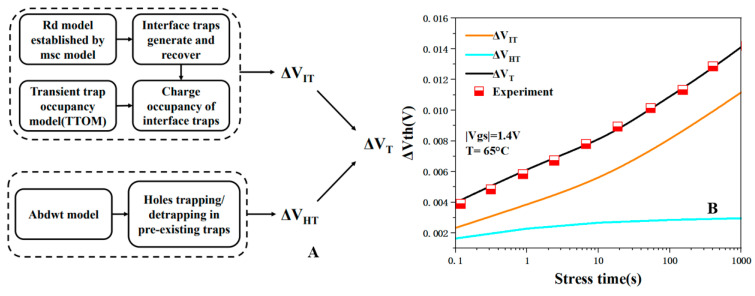
Schematic of NBTI modeling framework consisting of irrelevant ΔVIT and ΔVHT components (**A**), Calibration of NBTI framework(**B**), data from [37].

In order to describe the degradation of NBTI accurately, we made appropriate simplifications based on [32], ignored VOT that was not obvious under conventional stress, and calibrated the simplified BAT framework [10], which allowed us to simulate the NBTI effect of NSFET. Device degradation is divided into two unrelated parts: interface traps, expressed as ΔVit, and hole traps, expressed as ΔVht. The generation of interface traps is described by the multi-state configuration (MSC)–hydrogen transport degradation model [33], and we constructed a dual-interface RD model [32] based on this model. We set up two state transitions at the Si/SiO_2_ interface and SiO_2_/HfO_2_ interface to simulate the generation of traps. However, not all traps generated contribute to device degradation. Only interface traps above the Fermi level contribute to degradation. Therefore, we use the TTOM model to calculate the occupancy probability of interface traps to obtain the value of ΔVIT [32]. Hole trapping is simulated by the ABDWT model, which was first proposed in [34] and covered in recent reports [35,36]. Overall, ΔVT is calculated from two uncorrelated components, ΔVIT and ΔVHT. As shown in Figure 3, we verified the accuracy of this framework through the experimental data [37]. Since ΔVIT dominates ΔVT under actual stress conditions, only ΔVIT is considered in the following degradation simulations [38].

## 3. Results and Discussion

### 3.1. NBTI under the Influence of Self-Heating Effect

**Figure 4:**
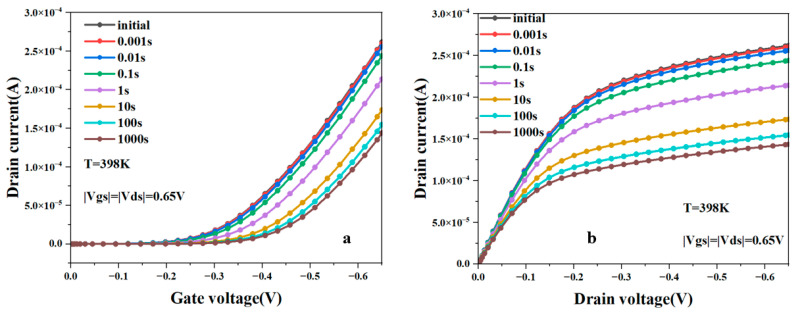
The transfer characteristic (**a**) curve and output characteristic curve (**b**) of NSFET change with the change in degradation time.

Figure 4a shows the change in the transfer characteristic curve of NSFETs with degradation time under the influence of the NBTI effect. It can be seen from the figure that as the degradation time increases, the drain current decreases, and the threshold voltage becomes larger. Figure 4b shows that the output characteristic curve of NSFET is affected by the NBTI effect. It can also be concluded from the figure that the drain current decreases with increasing degradation time. Table 2 shows the changes in the threshold voltage of NSFET before and after being affected by the NBTI effect. We can see from the table that the threshold voltage increases with the increase in degradation time.

**Figure 5:**
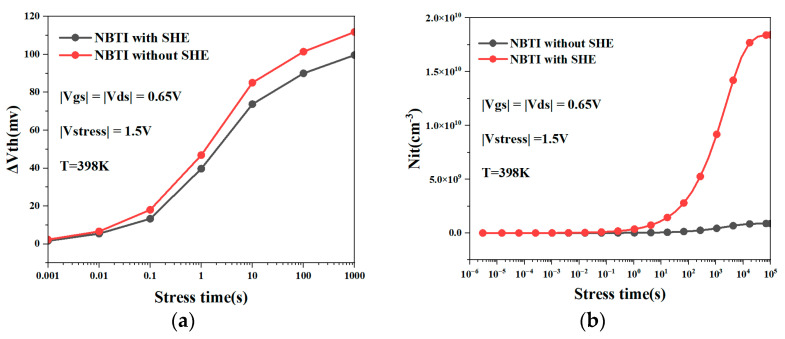
Under the influence of the self-heating effect, the threshold voltage degradation of the NSFET by NBTI is compared with that of the NSFET only affected by NBTI (**a**); interface traps of NSFET with or without self-heating (**b**).

Figure 5a compares the pure NBTI and NBTI effects under the self-heating effect of the NSFET. It is evident from the figure that self-heating will exacerbate the NBTI effect. Figure 5b shows the changes in the interface trap of the NSFET with and without the influence of self-heating as the degradation time increases. It can be observed from the figure that as the degradation time increases, the number of interface traps of the NSFET also increases. Under the joint influence of self-heating and NBTI effects, the number of interface traps in the NSFET is greater than that of NBTI alone. This is because the self-heating effect causes the temperature in the channel to increase, and the carriers gain more energy during transport. As a result, more carriers cross the interface barrier to form traps, generating more dangling bonds, further aggravating device degradation and causing the NBTI effect to become more serious.

**Figure 6:**
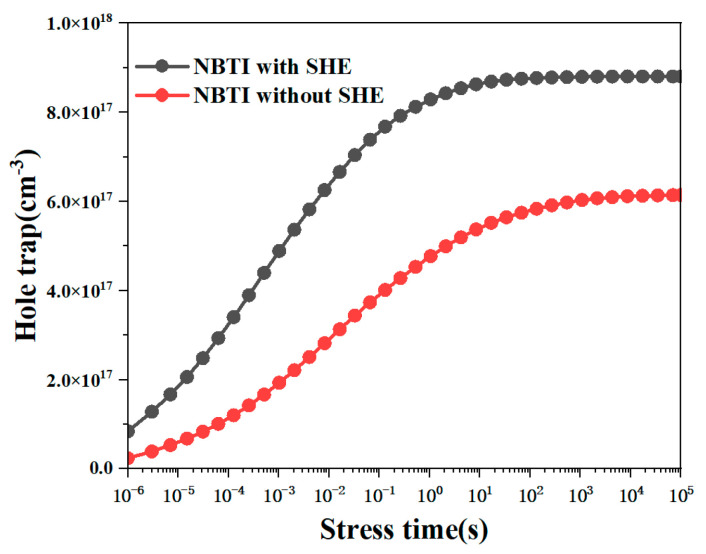
Changes in hole traps under self-heating effect.

Figure 6 shows the impact of the self-heating effect on hole traps, which reflects the change in ΔVHT value. It can be seen from the figure that under the influence of the self-heating effect, the number of hole traps increases and ΔVHT increases, which in turn leads to an increase in the degradation of ΔVT.

**Figure 7:**
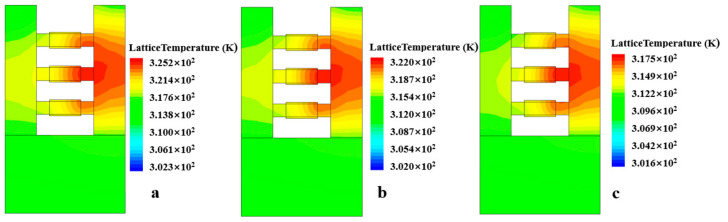
Schematic of NSFET under SHE, showing the lattice temperature is higher near the drain side than near the source side. Stress time = 0.001 s (**a**), stress time = 10 s (**b**), stress time = 10,000 s (**c**).

**Figure 8:**
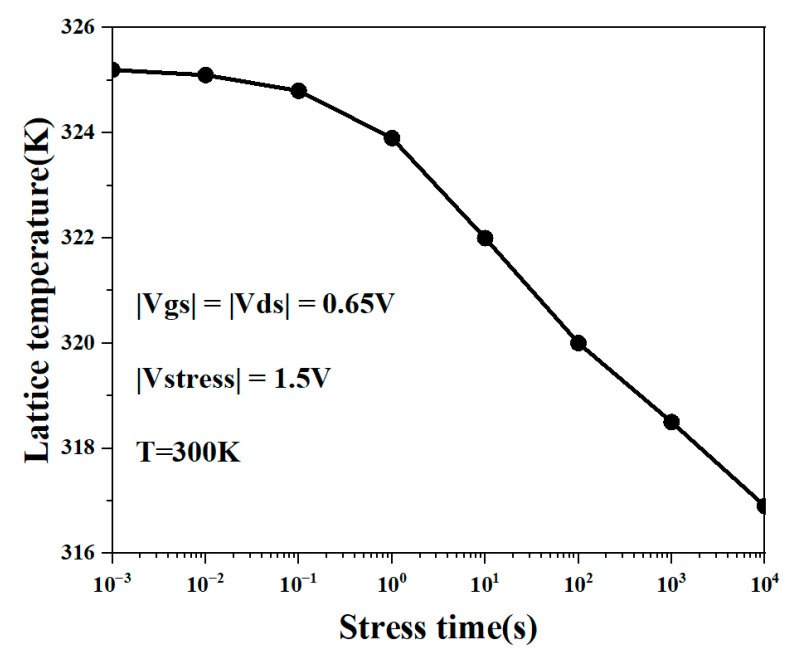
Lattice temperature of NSFET changes with NBTI degradation time.

Figure 7 shows the three-dimensional thermal distribution diagram of the NSFET under the influence of the NBTI effect. It can be observed from the figure that the heat generated by the device due to the self-heating effect is mainly distributed near the drain. This is mainly because carriers obtain energy through the electric field when transporting in the channel. Near the drain, the carriers move for the longest time, so the energy is higher, resulting in more thermal effects. In addition, we can also see that the thermal effect of the device gradually weakens as the degradation time increases. As the degradation time increases, more Si-H bonds will be broken under the action of holes, generating more dangling bonds and further aggravating the NBTI effect of the device. However, the number of carriers that react with Si-H bonds increases, and the number of carriers that reach the drain and exhibit thermal effects decreases. Hence, the self-heating effect of the device becomes less noticeable. Therefore, the NBTI effect weakens the self-heating effect. We can also conclude in Figure 8 that as the degradation time increases, the lattice temperature of the NSFET decreases.

These simulation results show that in nanoscale P-NSFETs, the self-heating effect will intensify the NBTI effect, and the NBTI effect will weaken the self-heating effect. This means that the interplay of these two effects needs to be balanced when designing and optimizing such nanodevices. By controlling the temperature and voltage of the device, the impact of NBTI can be effectively reduced while minimizing the negative impact of self-heating on device performance.

### 3.2. Effect of Nanosheet Width on NBTI and SHE

In NSFETs, compared to planar FETs, the electric field distribution in the channel area is not uniform because the channel is a thin sheet structure and the gate surrounds the channel. Therefore, there is an electric field enhancement effect in the channel of NSFETs, especially in the corner region. The electric field enhancement effect improves the gate control performance of the device to a certain extent [31], but it also aggravates the impact of the BTI effect. This means that in the same layer of the nanosheet, the distribution of the electric field is uneven, causing the BTI effect to have varying degrees of impact at different locations.

**Figure 9:**
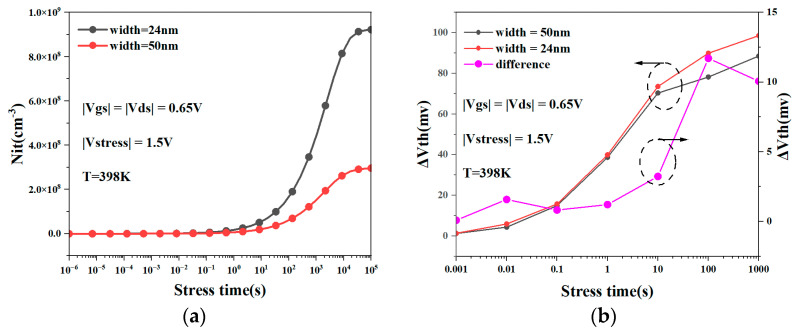
Interface traps of NSFETs with different nanosheet widths change with degradation time (**a**); NBTI degradation of NSFET at different nanosheet widths (**b**).

Figure 9a compares the changes in the interface traps of the NSFET with different nanosheet widths as the degradation time increases. We can see from the figure that as the degradation time increases, the NSFET with a wider nanosheet width has fewer interface traps than the NSFET with a narrower width. Figure 9b shows the NBTI degradation of the NSFET at different nanosheet widths. It can be observed from the figure that as the width of the nanosheet increases, the amount of NBTI degradation of the NSFET decreases. This demonstrates that the width of the nanosheet has a certain influence on the extent of NBTI degradation. This is because wider nanosheets are more conducive to uniform electric field distribution, and the electric field at the rolled corners is not exceptionally concentrated. The NBTI effect depends on the strength of the electric field. Reducing the electric field will alleviate the NBTI effect to a certain extent. Therefore, increasing the width of the nanosheet will weaken the NBTI effect of the NSFET. 

**Figure 10:**
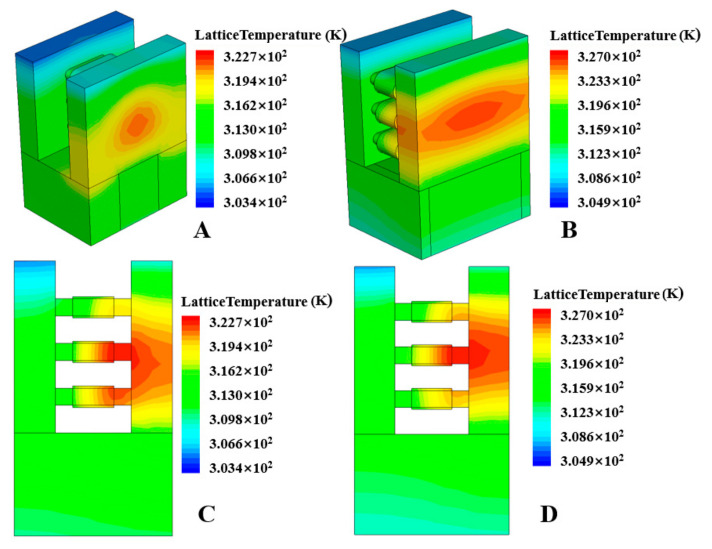
Schematic of NSFET with different nanosheet widths, showing lattice temperature. Width = 24 nm (**A**), width = 50 nm (**B**). Lattice temperature at the channel, width = 24 nm (**C**), width = 50 nm (**D**).

**Figure 11:**
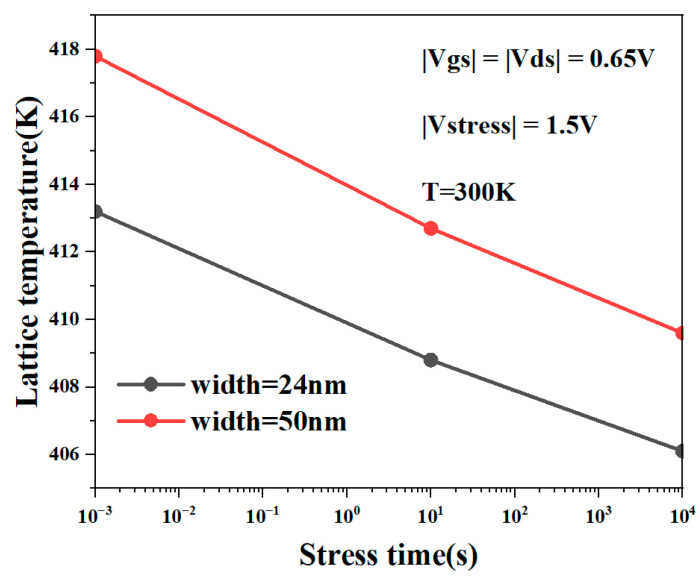
Lattice temperature of NSFET at different nanosheet widths changes with NBTI degradation time.

Figure 10A,B show the three-dimensional lattice temperature distribution of the device when the nanosheet width is 24 nm and 50 nm. It can be observed that as the nanosheet width increases, the heat distribution range of the NSFET expands. Figure 10C,D show the energy distribution at the channel. We can also conclude from the figures that the increase in width will increase the self-heating effect of the NSFET. The results show that increasing the nanosheet’s width will increase the device’s self-heating effect. The main reason is that as the width of the nanosheet increases, the number of carriers in the channel also increases, which causes more carriers to gain energy and collide with the lattice, which in turn causes the energy of the lattice to rise more, further aggravating the device’s self-heating effect. Figure 11 shows the lattice temperature of NSFET as a function of degradation time at different nanosheet widths. It can be seen from the figure that wider nanosheets have more significant lattice temperatures.

**Figure 12:**
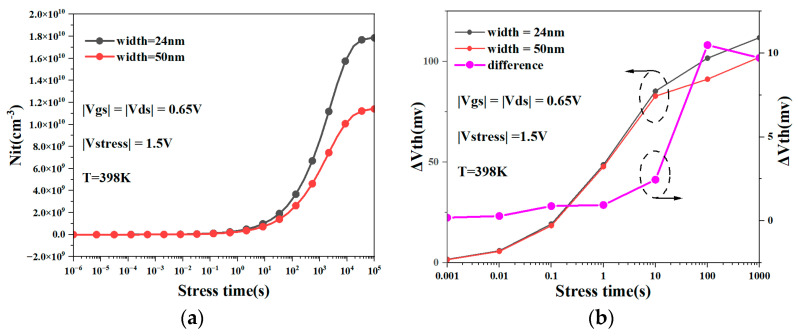
Interface traps under coupling effect of NSFET with different nanosheet widths (**a**); coupling effect of NBTI and self-heating on threshold voltage degradation of NSFETs with different widths (**b**).

Figure 12a shows the change in the number of interface traps as the degradation time increases for NSFETs with different nanosheet widths under the coupling influence of the self-heating effect and NBTI. We can see from the figure that under the same degradation time, the coupling effect of self-heating and NBTI has less impact on the interface traps of the NSFET with wider nanosheet width. Figure 12b shows the coupling effect of self-heating and NBTI on the threshold voltage degradation of the NSFET under different nanosheet widths. It can be observed from the figure that as the width of the nanosheet increases, the degradation caused by the coupling effect of the NSFET decreases. The results in Figure 9 and Figure 11 show that as the nanosheet width increases, the NBTI effect weakens while the self-heating effect increases. A comprehensive analysis of Figure 12 shows that in increasing the nanosheet’s width, the impact of the NBTI effect on the device is more significant than the impact of the self-heating effect on the device. 

### 3.3. Influence of NBTI and SHE on SRAM

**Figure 13:**
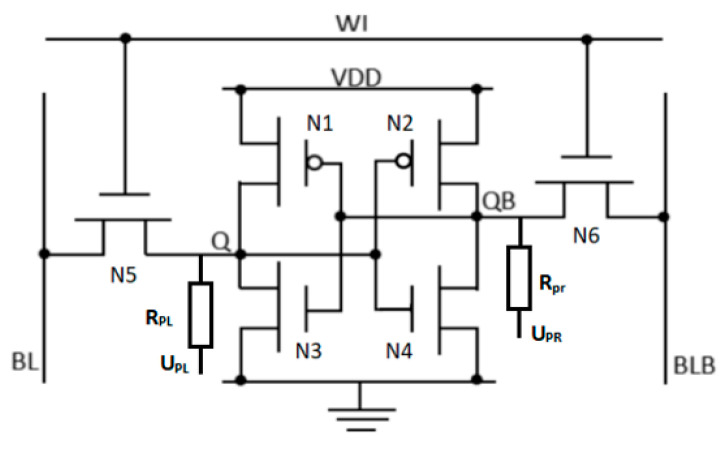
Constructing the circuit diagram of a 6T SRAM cell in Sentaurus TCAD.

In order to study the impact of NBTI and self-heating effects on SRAM performance, we built an NSFET-based SRAM circuit in Sentaurus TCAD. Figure 13 shows the structure of a 6T SRAM cell.

Stability is crucial in SRAM design. Usually, we use SNM (static noise margin) to evaluate the stability of the SRAM. SNM defines the maximum noise level an SRAM cell can tolerate while keeping its stored data error-free. Generally speaking, we measure the SNM of SRAM through the butterfly curve [39]. Depending on the working state of the device, the static noise margin of the SRAM unit circuit is divided into hold static noise margin (HSNM), read static noise margin (RSNM), and write static noise margin (WSNM). In SRAM, read operations are usually more frequent than write operations. The RSNM value is the smallest for the six-tube SRAM structure, so the RSNM value is often used as the static noise margin of the six-tube SRAM cell structure [40].

**Figure 14:**
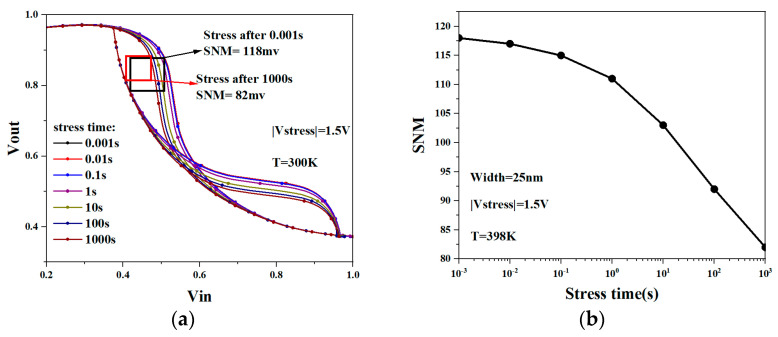
Butterfly curve of NSFET-based SRAM under NBTI effect (**a**); SNM degradation of NSFET-based SRAM under NBTI effect (**b**).

Figure 14 shows the butterfly curve of NSFET-based SRAM. It can be observed from the figure that as the degradation time increases, the SNM of SRAM gradually decreases. Under the influence of the NBTI effect, the two voltage output characteristic curves in the butterfly curve shift when the level flips, causing the level information of the corresponding storage node to be more susceptible to noise and flipping. This is because in 6T SRAM cells, PMOS acts as a pull-up transistor, and its threshold voltage is reduced due to the influence of NBTI, making it harder to turn on. At the same time, NMOS, as the pull-down transistor of 6T SRAM, is unaffected. When the potential of the Q point changes from logic 1 to logic 0, the PMOS turns on relatively late to charge the QB node, while the NMOS typically remains turned on. Therefore, as the degradation time increases, the butterfly curve in SRAM flips earlier.

**Figure 15:**
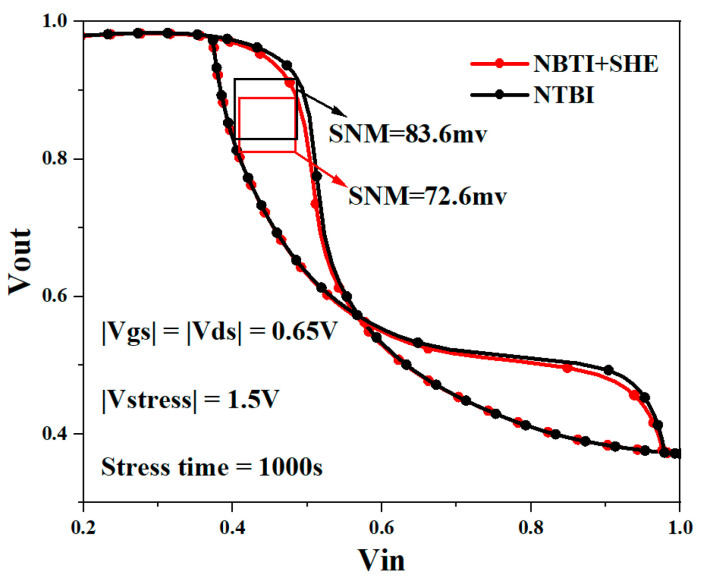
Butterfly curve of NSFET-based SRAM under NBTI and couple effect.

Figure 15 shows the butterfly curve of an NSFET-based SRAM cell under the influence of the self-heating effect and NBTI. It can be observed from the figure that under the same degradation time, the self-heating and NBTI coupling effects produce a more significant shift in the butterfly curve of the SRAM cell. According to the analysis in Section 3.2, self-heating and NBTI coupling effects cause more significant degradation in the threshold voltage of P-type NSFETs. Therefore, the P-type NSFET, the pull-up transmission gate of the SRAM, is less likely to be turned on, resulting in a more significant shift in the voltage output characteristic curve.

**Figure 16:**
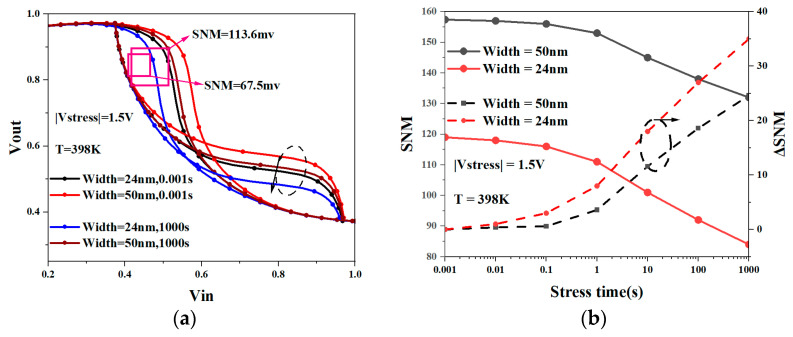
Butterfly curve of SRAM based on NSFET with different width nanosheet (**a**); SNM degradation of NSFET-based SRAM with different-width nanosheets (**b**).

Figure 16a shows the butterfly curve of SRAM based on NSFETs with different nanosheet widths under the influence of the NBTI effect. It can be seen that the butterfly curve shift of the SRAM constructed from NSFETs with a wider nanosheet width is smaller. Figure 16b shows the SNM degradation of NSFET-based SRAM with different-width nanosheets. The result shows that SRAM based on NSFETs with a wider nanosheet width has higher SNM and smaller SNM degradation. This is also because the wider nanosheet width helps the electric field to be distributed more evenly, thereby mitigating the impact of the BTI effect on the rolled corner area to a certain extent. However, this also increases the SRAM cell area, requiring designers to trade between integration and reliability.

## 4. Conclusions

In nanoscale P-NSFET technology, SHE significantly increases the impact of NBTI, but as the NBTI degradation time increases, the self-heating effect weakens. In addition, due to the uneven electric field distribution in the nanosheets, there is an electric field enhancement effect in the rolled corner area, which leads to increased NBTI degradation of the device. Increasing the width of the nanosheet can reduce the proportion of the rolled corner area in the nanosheet, making the electric field distribution more uniform, thereby weakening the impact of NBTI on the device. We also extend our research to 6T SRAM cell circuits. As the NBTI degradation time increases, the NBTI effect will reduce the SNM of SRAM, and the coupling effect of NBTI and self-heating will aggravate the SNM degradation. At the same time, we demonstrated that increasing the nanosheet width weakens the degradation of SNM. However, increasing the nanosheet’s width will increase the SRAM cell’s area, which requires designers to make a trade-off between the integration and reliability of the unit circuit.

## Figures and Tables

**Table 1:** Device parameter for the 5 nm node NSFET.

Symbol	Implication	Value
Lg	Gate length	12 nm
Lext	Extension length	5 nm
Ls/d	S/D length,	12 nm
Tch	Channel height	5 nm
Tw	Channel width	25 nm
Nc	Channel doping concentration	1 × 10^16^ cm^−3^
Nd	Drain doping concentration	1 × 10^20^ cm^−3^
Ns	Source doping concentration	1 × 10^20^ cm^−3^
Eot	Equivalent oxide thickness	0.9 nm
Vth0	Initial Vth before NBTI effect	−0.3792 V

**Table 2:** NSFET threshold voltage changes with degradation time.

Aging Time	Initial	NBTI Aging
0.001 s	−0.3729 V	−0.3746
0.01 s	−0.3729 V	−0.3784
0.1 s	−0.3729 V	−0.3862
1 s	−0.3729 V	−0.4126
10 s	−0.3729 V	−0.4467
100 s	−0.3729 V	−0.4629
1000 s	−0.3729 V	−0.4726

## Data Availability

All data that support the findings of this study are included within the article.
